# Chromophobe renal cell carcinoma with sarcomatoid and heterologous osteosarcoma-like differentiation: a case report and literature review

**DOI:** 10.1093/jscr/rjad476

**Published:** 2023-08-23

**Authors:** Saleh Abuorouq, Firas Sahawneh, Omar Halalsheh, Husam K Haddad, Raed Mekail, Hiba Alzoubi, Mo’ath Alrjoub, Hashem A Serhan

**Affiliations:** Department of Clinical Medical Sciences, Urology Division, Faculty of Medicine, Yarmouk University, Irbid 21163, Jordan; Private Sector, Department of Urology, Irbid 21110, Jordan; Department of Urology, Faculty of Medicine, Jordan University of Science and Technology, Irbid 22110, Jordan; Department of Pathology and Laboratory Medicine, Ministry of Health, Amman 11118, Jordan; Private Sector, Department of Urology, Irbid 21110, Jordan; Department of Basic Medical Sciences, Pathology Division, Faculty of Medicine, Yarmouk University, Irbid 21163, Jordan; Department of Pathology and Microbiology, Faculty of Medicine, Jordan University of Science and Technology, Irbid 22110, Jordan; Department of Ophthalmology, Hamad Medical Corporations, Doha 3050, Qatar

**Keywords:** chromophobe, renal cell carcinoma, osteosarcoma, sarcomatoid, differentiation

## Abstract

Chromophobe renal cell carcinoma (CRCC) is a subtype of renal cell carcinoma (RCC) with a favorable prognosis. Sarcomatoid differentiation in RCC is assumed to be the outcome of the parent tumor’s dedifferentiation and associated with poorer prognosis. Sarcomatoid differentiation can be detected in CRCC as well as other subtypes, but the occurrence of divergent osteosarcoma-like components in sarcomatoid CRCC is extremely unusual. Only six cases have been previously reported in the literature, we reviewed them and presented the seventh case in a 71-year-old male who had a left kidney heterogeneous mass. The resected tumor showed a sarcoma-like spindle cell area with an adjacent osteosarcoma area producing lacy bone material and bony trabeculae in a hard area mixed with a typical CRCC. In conclusion, sarcomatoid CRCC with osteosarcomatous differentiation is a very rare tumor and should be kept in mind especially when dealing with small or frozen sections biopsies.

## INTRODUCTION

Chromophobe renal cell carcinomas (CRCC) are an uncommon type of renal cell carcinoma (RCC) that accounts for around 5% of all RCC cases [1]. In general, CRCC has a better prognosis than other RCC subtypes [1]. Sarcomatoid change in RCC occurs when a parent tumor dedifferentiates into a higher-grade malignancy and it is typically characterized by a big size, poor prognosis, and significant cytological atypia, with highly atypical spindle cells resembling a sarcoma [2, 3]. Sarcomatoid dedifferentiation can happen in any kind of RCC, including clear-cell carcinoma, papillary RCC, collecting-duct carcinoma, and in CRCC [2]. The occurrence of heterologous osteosarcoma-like components in sarcomatoid CRCC is extremely unusual [4–9]. We present the seventh case of this rare entity of osteosarcoma component arising in sarcomatoid CRCC, and review of the previous six cases reported in the literature [4–9], with a thorough discussion of the main differential diagnosis of such masses.

## CASE REPORT

**Figure 1:**
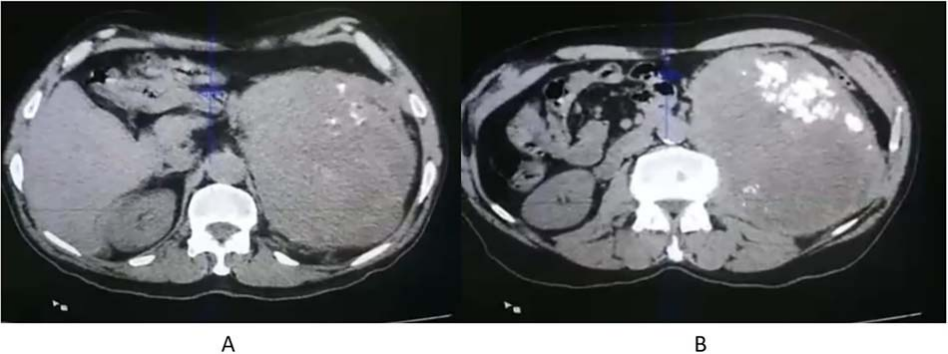
CT scan axial (**A**, **B**) showing left renal solid mass, disrupting the upper two-third of renal parenchyma, with thick dystrophic calcifications (**B**).

A 71-year-old Jordanian male with a known case of hypertension presented with left flank pain for 3 months. An abdominal computed tomography (CT scan) revealed a huge, 20 × 12 × 7 cm left renal solid mass, disrupting the upper two-third of renal parenchyma, with thick dystrophic calcifications, and causing displacement of the surrounding bowel structure (Fig. 1). Clinically, a renal tumor was suspected and left nephrectomy was performed. The patient recovered well after the operation without any surgical complications.

The lesion was immediately fixed in a 10% formalin-buffered solution for 48 h after surgical excision and embedded in paraffin. The paraffin tissue blocks were divided into 5-μm sections and stained with hematoxylin and eosin. Additional 5-μm histological sections were kept for the immunohistochemical analysis. Grossly, the resected specimen consisted of the left kidney, weighing 2100.0 grams and measuring 20.0 × 11.0 × 7.0 cm with an attached ureter measuring 4.0 × 0.7 × 0.7 cm. Serial sectioning shows a heterogeneous mass lesion involving the whole kidney measuring 20.0 × 16.0 × 12.0 cm. The mass lesion is necrotic and friable with about 50% showing necrosis and focally the tumor was so hard that it could not be cut. The mass lesion has pushing peripheral margins with a lobulated appearance and invading the perinephric and sinus fat.

**Figure 2:**
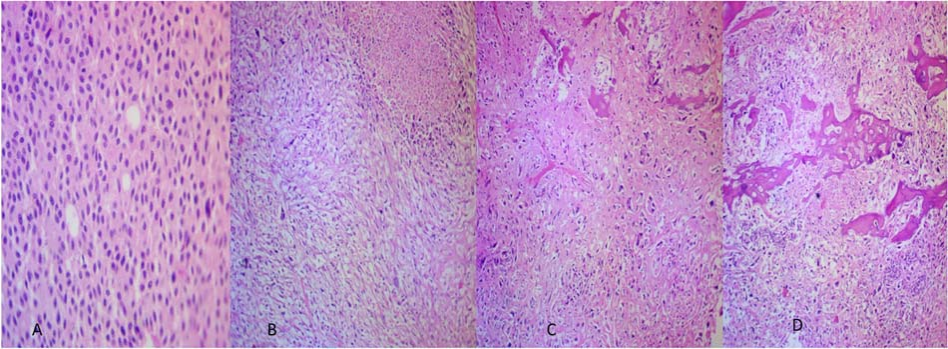
(**A**) The CRCC area (×40). (**B**) Sarcomatoid areas showing atypical lacy mineralized bone material and large pleomorphic spindle cell proliferation with necrosis (×40). (**C**, **D**) Osteosarcomatous areas showing atypical lacy mineralized bone material, and atypical spindle cells existing between the osteoid (×40).

**Figure 3:**
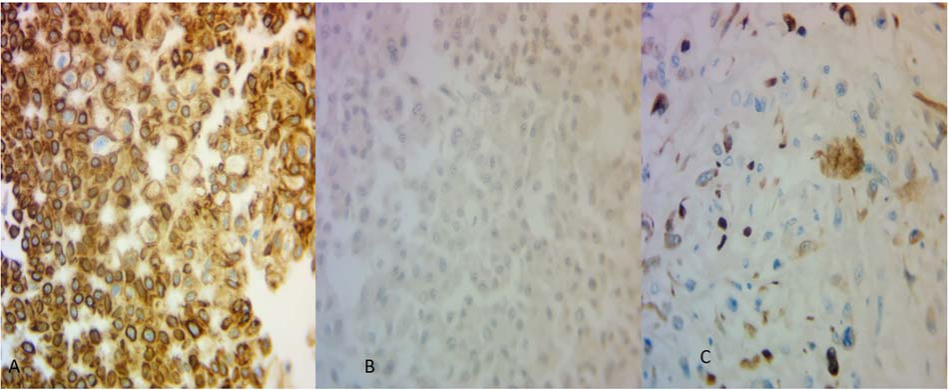
(**A**) The areas of CRCC were positive for CK7 (×40) and (**B**) negative for CA-IX (×40). The osteosarcoma-like areas were focally and strongly positive for CK AE1/AE3 (×40).

The microscopic examination of the kidney tumor showed a biphasic pattern composed of 10% of classic malignant chromophobe epithelial components mixed with the high-grade sarcomatoid neoplasm (50%; Fig. 2A and B) intermingled with areas showing atypical lacy mineralized bone material and focal well-formed bony trabeculae were also identified (Fig. 2C and D). The chromophobe component showed polygonal cells with centrally located round hyperchromatic nuclei, pale, granular, eosinophilic cytoplasm, and faint cytoplasmic borders (Fig. 2A). The sarcomatoid area shows large, pleomorphic, mitotically active spindle cells, and extensive necrosis (Fig. 2B). The tumor cells in the chromophobe are strongly and diffusely positive for Cytokeratin 7 (CK7) and negative for Carbonic anhydrase IX (CA-IX; Fig. 3A and B). Additionally, the sarcomatoid area shows focal strong positivity for CK AE1/AE3 (Fig. 3C). The focal immunohistochemical expression of cytokeratin (Fig. 3C) by the sarcomatoid cells along with the close relation with a pure CRCC led us toward the diagnosis of sarcomatoid CRCC with osteosarcomatous differentiation.

## DISCUSSION

CRCC constitutes about 5% of surgically resected renal epithelial tumors [1]. The average age of patients with CRCC is 60 years, and the mortality rate is <10% [1]. CRCC has a low malignant potential and a favorable prognosis [10]. However, it is a highly aggressive tumor when it appears with sarcomatoid differentiation or necrosis of the sarcomatoid region [2]. The disease-specific survival rate for RCC with sarcomatoid transformation was 22% in the study of de Peralta-Venturina *et al*. [2], which is lower than that of RCC without sarcomatoid differentiation (79%).

Sarcomatoid differentiation is common in CRCC, accounting for 9% of all RCC subtypes. de Peralta-Venturina *et al*. investigated 100 kidney tumors and discovered that sarcomatoid differentiation occurred at a rate of 8% in conventional RCC, 9% in chromophobe RCC, 3% in papillary RCC, 29% in collecting duct carcinoma, and 11% in unclassified RCC [2, 11]. The presence of a sarcomatoid component has been linked to an increased risk of metastasis and a poor prognosis [2, 5, 11]. Moreover, the typical 5-year disease-specific survival rate of CRCC is close to 100%, with a progression-free survival rate of 94%, and drops to levels equivalent to other sarcomatoid RCCs. In most cases, the dedifferentiated sarcomatoid component, are homologous in type; malignant fibrous histiocytomas (44%), fibrosarcomas (53%), and 3% are unclassified sarcomatoid tumor, but the occurrence of heterologous osteosarcoma is extremely rare [2]. The sarcomatoid component’s proportion may vary from case to case. However, de Peralta Venturina *et al*. study found no significant correlation between cases with a high and low percentage of sarcomatoid regions, and TNM stages are the sole independent predictors of survival.

Only six cases of osteosarcoma-like differentiation in sarcomatoid CRCC have been reported in the literature [4–9]. Table 1 summarizes the primary clinical and pathologic aspects of these cases as well as the present case. Most of the cases of CRCC with osteosarcoma differentiation were diagnosed in patients in their seventh decade, which is older than the average age at which CRCC without osteosarcoma differentiation is usually diagnosed [7]. More than 50% of the cases showed a high percentage of sarcomatoid components and were associated with extensive necrosis. Most of these patients presented with frequent regional or distant metastases and short survival as shown in Table 1.

**Table 1:** Review of the literature on sarcomatoid CRCC with osteosarcomatoid differentiation.

Authors (YOP)	Ethnicity	Age	Gender	Kidney	Presentation	CT scan	Metastasis	Management	Histopathological findings	Outcome	Reference
Itoh T. *et al*. (2002)	Japanese	74	M	Right	Fatigue, anorexia, a huge palpable mass on the posterior hypochondriac region, and marked abdominal distention from ileus	Huge retroperitoneal mass with calcification, which destroyed the lower half of the right kidney and directly invaded the ascending and sigmoid colon	Multiple intra-abdominal dissemination and liver and pulmonary metastases	Nephrectomy and ascending colon resection because the tumor invaded the colon.	Chromophobe RCC with osteosarcoma-like differentiation (66%) and focal sarcomatoid area. Extensive necrosis.	Died 2 months after the diagnosis	[4]
Gira F. *et al*. (2008)	NA	73	F	Left	Fatigue, increasing abdominal pain, and hematuria	Heterogeneous left kidney mass (19 × 12 × 23 cm), relegated the kidney upper-medially into the retroperitoneum with focal calcification	Lung nodule	Nephrectomy	Chromophobe RCC (10%) with sarcomatoid dedifferentiation and osteosarcoma-like divergent differentiation. Extensive necrosis and hemorrhage	Died shortly after the operation as a consequence of the disseminated disease	[5]
Quiroga G. *et al*. (2009)	NA	63	F	Left	Episodes of nausea and mild left flank pain for 2 months	Upper pole left renal mass with renal vein involvement	Multiple pulmonary nodules, along with hepatic and bone (sacral) metastases.	Left radical nephrectomy with a periaortic lymph node dissection, as well as left adrenalectomy and splenectomy and radiotherapy and chemotherapy for distant metastatic disease	Chromophobe RCC with sarcomatoid dedifferentiation (70%) and 5% of osteosarcomatous and chondrosarcomatous components. Tumor necrosis (40%).	Alive for the 10 months after the diagnosis	[6]
Li YF *et al*. (2010)	Taiwanese	78	F	Right	Fatigue, anorexia, and a huge palpable mass in the posterior hypochondriac region	Huge, ill-defined heterogeneous kidney mass 14.2 × 14.5 × 15.9 cm and several adjacent enlarged lymph nodes	None	Nephrectomy	Chromophobe RCC with sarcomatoid- like differentiation	Alive 6 months after diagnosis	[7]
Sari A. *et al*. (2011)	NA	49	NA	Left	Abdominal pain for 2 months	Solid mass of 25 × 20 cm in size that extended from the level of the left renal hilus to the iliac wing origin with a regular outer contour and necrosis foci	None	Nephrectomy	Sarcomatoid CRCC with osteosarcomatous differentiation	Died because of surgical complications	[8]
Bharti S (2020)	NA	64	F	Left	Palpable mass in the lower left abdomen and hematuria	Heterogeneous peripherally enhanced tumor with a central hypovascular area	Lymph node metastasis	Left radical nephrectomy	Chromophobe RCC with sarcomatous differentiation containing osteosarcoma component	NA	[9]
The present case	Jordanian	71	M	Left	Left flank pain for 3 months	Huge, 20 × 12 × 7 cm left renal solid mass, disrupting the upper two-third of renal parenchyma, with thick dystrophic calcifications, and causing displacement of the surrounding bowel structure	None	Nephrectomy	CRCC (10%) with sarcomatoid and osteosarcoma-like differentiation. Extensive necrosis	Alive one month after the operation	

Abbreviations: YOP, year of publications; F, female; M, male; NA, not applicable.

Regarding CRCC, it has recently been demonstrated that the epithelial and sarcomatoid components of chromophobe RCC have different genetic abnormalities than pure chromophobe RCC, implying that multiple chromosomal gains are important factors in the sarcomatoid transformation [12]. According to Akhtar *et al*. [3], the development of sarcomatoid cells in CRCC may be connected with its unique genetic profile, which makes the cells prone to hyperploidization. Brunelli *et al*. [12] conducted considerable research on this result, discovering that both the epithelium and sarcomatoid elements in a CRCC exhibit different genetic anomalies. Multiple gains of Chromosomes 1, 2, 6, 10, and 17.15 are seen in sarcomatoid CRCC. Additionally, Quiroga-Garza *et al*. [6] reported a CRCC case with sarcomatoid differentiation, as well as a metastatic periaortic lymph node tumor with only a sarcomatoid component. These findings lend credence to the existence of a tumor progression pathway from chromophobe to sarcomatoid RCC. However, the precise sequence of genetic events involved in this change is still unknown.

Reporting such cases is very important, especially when dealing with retroperitoneal small biopsies or frozen section biopsies, which make the differential diagnosis more challenging. This is especially crucial when this biopsy shows the osteosarcomatous component, which could either be originating from the kidney as in our case, or secondarily involving the kidney. The differential diagnosis approach for such cases included extraskeletal osteosarcoma in the retroperitoneum or sarcomatoid RCC with osteosarcomatous differentiation. Extraskeletal osteosarcoma, on the other hand, is most commonly found in the lower extremities (46.6%), upper extremities (20.5%), or the retroperitoneum (17%) [7, 13, 14]. In our case, the synchronous presence of well-differentiated chromophobe and dedifferentiated sarcomatoid components, the transition between these areas, and the focal cytokeratin positivity in the sarcomatoid areas were used to make the diagnosis of sarcomatoid RCC, and excluding extraskeletal osteosarcoma of the retroperitoneum.

In summary, we report a case of sarcomatoid CRCC with osteosarcoma differentiation that is apparently the seventh reported case of heterologous sarcomatoid CRCC. However, because of the rarity of this occurrence, the exact relationship between the existence of heterologous osteosarcomatous components, in particular, and prognosis remains unknown. Additionally, this rare tumor should be taken into consideration when dealing with small biopsies of retroperitoneal masses or frozen sections biopsy of retroperitoneal or kidney masses.

## Data Availability

All data generated or analyzed during this study are included in this published article.
